# Supplementary material for: The Link between Three Single Nucleotide Variants of the GIPR Gene and Metabolic Health
Source: Genes (Basel). 2022 Aug 26;13(9):1534. doi: 10.3390/genes13091534 (PMC9498707; doi:10.3390/genes13091534)
Supplement: Supplementary file 1 [file genes-13-01534-s001.zip › genes-1885129-supplementary.pdf]

SUPPLEMENTARY MATERIALS

Table S1

HRM parameters and primer sequences for genotyping of rs11672660. rs2334255 and rs10423928 variants

SNV	Chromosome position	Alleles	Primers for PCR amplification (5′–3′)	Annealing Temp. (°C)	Melt. Temp. Range (°C)
**rs11672660**	chr19: 45676926	C/T	Left: CGGGCAGCGCTGACTACC Right: GTAGTTGGCACCCACGCAGT	58	84–96
**rs2334255**	chr19: 45682892	G/T	Left: CTCAAGTGATCCACCCACCT Right: CCTGCAGTCCCCTCCTTT	60	82-92
**rs10423928**	chr19: 45679046	T/A	Left: GTTTACCTGAGTCCCCAACCT Right: TGTGATAAGCCTGGAGGAGTG	61	79-89

HRM. high-resolution melting curve analysis; SNV. Single Nucleotide Variant; PCR. polymerase chain reaction

Table S2

Comparison of anthropometric measurements and metabolic parameters between particular genotypes of rs11672660 variant in the whole study population

rs11672660	TT			CT			CC			p
	Mean	Median	SD	Mean	Median	SD	Mean	Median	SD	
Age [years]	53.71	5750	13.44	53.53	57.00	14.34	53.39	55.00	14.34	0.9714
Body mass [kg]	74.39	69.20	15.18	74.88	72.85	15.70	75.01	73.70	15.17	0.8488
BMI [kg/m^2]	26.88	25.88	4.91	26.85	26.25	4.84	27.23	26.70	5.04	0.4849
WC [cm]	90.68	88.00	14.10	92.22	91.00	14.66	92.39	92.00	14.50	0.5457
NC [cm]	36.49	36.00	3.62	36.40	36.00	3.97	36.27	35.75	3.75	0.8808
Glucose [mg/dl]	97.22	90.00	21.43	95.98	91.00	26.44	95.12	90.00	23.88	0.5887
TG [mg/dl]	156.41	126.00	102.15	153.77	128.00	106.24	147.59	120.00	113.66	0.3948
HDL [mg/dl]	67.43	61.00	19.46	64.51	63.00	16.87	64.71	62.00	17.19	0.8273
AST [IU/l]	28.54	26.50	9.03	28.51	25.00	13.48	28.26	26.00	10.62	0.5251
ALT [IU/l]	31.78	25.00	20.33	31.47	26.00	20.47	31.43	26.50	21.99	0.9812

SD: standard deviation; BMI: Body Mass Index; WC: waist circumference; NC: neck circumference; TG: triglycerides; HDL: high density lipoprotein; AST: aspartate transaminase; ALT: alanine transaminase

Table S3

Comparison of anthropometric measurements and metabolic parameters between particular genotypes of rs10423928 variant in the whole study population

rs10423928	AA			AT			TT			p
	Mean	Median	SD	Mean	Median	SD	Mean	Median	SD	
Age [years]	52.80	56.00	13.33	53.85	57.00	14.37	53.59	56.00	14.33	0.8407
Body mass [kg]	73.74	69.10	15.18	74.87	72.83	15.64	75.37	74.00	15.13	0.4873
BMI [kg/m^2]	26.40	25.70	5.01	26.85	26.20	4.83	27.28	26.80	4.99	0.1744
WC [cm]	90.00	88.00	14.57	92.30	91.00	14.61	92.78	93.00	14.49	0.2690
NC [cm]	36.72	36.25	3.69	36.41	36.00	3.96	36.37	36.00	3.76	0.8427
Glucose [mg/dl]	96.16	90.00	21.22	95.97	91.00	26.31	95.08	90.00	24.03	0.7915

TG [mg/dl]	173.59	136.00	124.03	152.71	127.00	104.32	144.92	118.00	111.03	0.1684
HDL [mg/dl]	65.80	61.00	17.85	64.36	63.00	16.99	64.67	62.00	17.10	0.9788
AST [IU/l]	28.79	26.00	9.40	28.50	25.00	13.37	28.42	26.00	10.83	0.4917
ALT [IU/l]	32.75	25.50	21.20	31.32	26.00	20.33	31.54	27.00	21.67	0.9221

SD: standard deviation; BMI: Body Mass Index; WC: waist circumference; NC: neck circumference; TG: triglycerides; HDL: high density lipoprotein; AST: aspartate transaminase; ALT: alanine transaminase

Table S4

Comparison of anthropometric measurements and metabolic parameters between particular genotypes of rs2334255 variant in the whole study population

rs2334255	TT			GT			GG			p
	Mean	Median	SD	Mean	Median	SD	Mean	Median	SD	
Age [years]	57.87	58.00	12.71	53.37	56.00	14.20	53.90	57.00	14.27	0.2090
Body mass [kg]	74.70	75.00	11.76	74.52	72.65	15.38	75.44	73.15	15.38	0.6602
BMI [kg/m^2]	26.54	26.64	4.07	27.04	26.10	5.11	27.15	26.59	4.92	0.8520
WC [cm]	91.70	95.00	11.72	91.87	91.50	14.89	92.90	92.00	14.54	0.6223
NC [cm]	36.32	36.00	3.29	36.28	36.00	3.82	36.51	36.00	3.85	0.7316
Glucose [mg/dl]	94.96	92.00	15.07	95.79	90.00	26.73	95.43	90.00	24.29	0.7094
TG [mg/dl]	123.65	112.00	67.24	158.91	120.00	140.79	146.04	124.00	90.53	0.3588
HDL [mg/dl]	60.95	60.00	17.91	65.23	63.00	17.56	64.59	62.00	16.91	0.2917
AST [IU/l]	25.52	24.00	5.27	28.56	26.00	13.36	28.58	26.00	11.00	0.2781
ALT [IU/l]	27.19	23.00	11.03	31.97	26.00	22.69	31.70	27.00	20.95	0.2843

SD. standard deviation; BMI. Body Mass Index; WC. waist circumference; NC. neck circumference; TG. triglycerides; HDL. high density lipoprotein; AST. aspartate transaminase; ALT. alanine transaminase

Table S5

Comparison of anthropometric measurements and metabolic parameters between particular genotypes of rs11672660 variant in the control and the study group, and the group with and without metabolic syndrome

**rs11672660**	TT			CT			CC			p
	Mean	Median	SD	Mean	Median	SD	Mean	Median	SD	
**Study group**										
Age [years]	56.97	61.00	12.18	56.74	60.00	13.01	56.61	60.00	13.54	0.9996
Body mass [kg]	80.15	76.75	15.58	82.44	82.00	13.61	82.33	81.00	13.02	0.5527
BMI [kg/m^2]	29.34	27.90	4.25	29.49	28.40	4.06	29.97	29.00	4.09	0.1319
WC [cm]	97.33	94.25	12.68	99.17	98.00	12.10	99.54	99.00	11.81	0.3025
NC [cm]	37.52	37.00	3.59	37.68	38.00	3.65	37.49	37.00	3.47	0.8541
Glucose [mg/dl]	99.27	90.00	25.26	99.67	93.00	30.49	97.74	91.50	26.66	0.8419
TG [mg/dl]	177.79	150.00	112.13	174.03	150.00	114.10	169.15	138.00	128.49	0.5071
HDL [mg/dl]	61.09	60.00	13.78	60.34	60.00	15.13	60.82	59.00	15.85	0.9427
AST [IU/l]	29.36	27.00	8.90	29.30	26.00	15.13	29.57	27.00	11.93	0.2691
ALT [IU/l]	36.06	28.00	22.97	34.21	28.00	22.11	35.57	29.00	25.17	0.7984
**Control group**										
Age [years]	47.56	41.50	13.88	48.24	48.00	15.16	47.95	46.00	14.13	0.9525
Body mass [kg]	63.51	64.25	5.34	62.09	60.00	9.52	61.94	60.10	8.46	0.4610
BMI [kg/m^2]	22.25	22.42	1.57	22.37	22.29	1.82	22.32	22.60	1.79	0.8949
WC [cm]	78.86	79.50	7.00	80.33	79.00	10.38	79.39	79.00	8.75	0.8578

NC [cm]	34.38	34.00	2.74	33.68	33.00	3.20	33.77	33.00	2.97	0.5372
Glucose [mg/dl]	93.25	90.00	10.26	89.27	87.55	15.59	90.33	87.00	17.03	0.2055
TG [mg/dl]	120.65	109.00	67.57	119.15	97.00	80.45	108.97	94.00	62.66	0.6575
HDL [mg/dl]	80.12	89.00	23.39	71.94	70.00	17.50	72.49	69.00	17.40	0.5138
AST [IU/l]	27.25	25.00	9.56	26.99	25.00	9.16	25.60	25.00	6.40	0.9380
ALT [IU/l]	23.44	20.00	10.28	26.03	21.50	15.42	23.11	22.00	8.90	0.5773
**Group with MS**										
Age [years]	62.83	63.00	10.43	59.84	62.50	11.54	59.88	63.00	12.49	0.5838
Body mass [kg]	78.49	75.75	13.62	80.80	80.20	14.93	82.50	80.85	14.94	0.4234
BMI [kg/m^2]	28.90	27.60	4.33	29.59	29.17	4.71	30.33	29.80	4.98	0.1691
WC [cm]	98.71	96.00	11.93	99.87	99.50	12.66	102.12	100.00	12.65	0.1605
NC [cm]	37.44	37.00	3.76	37.53	37.00	3.80	37.84	38.00	3.46	0.7012
Glucose [mg/dl]	109.89	106.00	30.09	107.41	100.50	34.95	106.51	97.00	31.75	0.7616
TG [mg/dl]	219.17	161.00	130.71	217.30	188.00	125.27	219.86	192.00	152.36	0.8041
HDL [mg/dl]	63.33	56.50	21.86	57.78	56.00	14.39	56.26	54.00	14.61	0.3454
AST [IU/l]	30.33	26.50	10.57	27.09	25.00	8.71	31.07	28.00	13.34	0.0042[1]
ALT [IU/l]	40.89	28.50	28.58	32.02	27.00	16.72	36.99	31.00	20.96	0.0456[1]
**Group without MS**										
Age [years]	48.84	43.00	12.55	49.17	50.00	14.70	51.05	52.00	14.25	0.4114
Body mass [kg]	72.43	68.00	16.42	70.63	67.50	15.60	71.21	69.35	13.60	0.6615
BMI [kg/m^2]	25.91	25.00	5.13	25.13	24.70	4.31	25.87	25.30	4.30	0.1500
WC [cm]	86.77	85.00	13.88	87.13	87.50	14.44	88.13	87.50	13.09	0.4510
NC [cm]	35.88	35.00	3.53	35.44	35.00	3.96	35.38	35.00	3.61	0.7910
Glucose [mg/dl]	90.04	89.00	9.30	87.11	86.00	11.83	88.62	87.00	15.08	0.2696
TG [mg/dl]	120.42	108.00	58.80	106.28	97.00	53.36	107.86	97.00	56.22	0.3894
HDL [mg/dl]	69.77	61.00	18.47	69.66	66.00	16.97	69.21	66.16	16.89	0.8474
AST [IU/l]	27.27	26.50	7.86	29.74	26.00	16.60	26.50	25.00	8.04	0.3583
ALT [IU/l]	25.57	23.00	9.72	31.08	25.00	23.51	28.04	24.00	21.13	0.4584

SD: standard deviation; BMI: Body Mass Index; WC: waist circumference; NC: neck circumference; TG: triglycerides; HDL: high density lipoprotein; AST: aspartate transaminase; ALT: alanine transaminase; MS: metabolic syndrome; Kruskal-Wallis test was performed and when $p<0.05$ post-hoc Dunn's test was applied. [1]The difference between CT and CC was shown for AST (p=0.0033) and ALT (p=0.0457)

Table S6

Comparison of anthropometric measurements and metabolic parameters between particular genotypes of rs2334255 variant in the control and the study group, and the group with and without metabolic syndrome

**rs2334255**	TT			GT			GG			p
	Mean	Median	SD	Mean	Median	SD	Mean	Median	SD	
**Study group**										
Age [years]	59.56	64.00	13.10	56.72	60.00	13.37	57.11	60.00	12.98	0.6032
Body mass [kg]	80.60	79.00	8.23	82.53	82.00	13.33	82.21	81.05	13.57	0.9122
BMI [kg/m^2]	28.47	27.50	3.15	30.06	29.40	4.15	29.66	28.60	4.10	0.0718
WC [cm]	96.98	98.00	8.33	99.42	99.00	12.26	99.66	98.75	12.06	0.7558
NC [cm]	37.52	38.00	2.77	37.75	37.75	3.56	37.57	38.00	3.56	0.8046
Glucose [mg/dl]	94.90	92.00	12.44	100.05	93.00	30.75	97.99	91.00	27.45	0.4999
TG [mg/dl]	132.00	119.00	70.98	189.55	151.50	164.39	163.23	141.50	94.62	0.1194

HDL [mg/dl]	58.60	57.00	19.46	60.28	59.00	15.21	60.85	60.00	15.25	0.4015
AST [IU/l]	25.00	23.00	5.18	30.38	27.00	15.61	29.37	27.00	11.88	0.1002
ALT [IU/l]	27.43	23.00	12.39	37.27	29.00	26.04	34.46	29.00	23.10	0.0485[1]
**Control group**										
Age [years]	54.64	55.00	13.37	48.28	48.00	14.04	48.24	46.00	14.85	0.3424
Body mass [kg]	61.29	62.00	6.05	62.07	60.55	8.56	62.54	60.50	9.10	0.9569
BMI [kg/m^2]	22.15	22.80	1.91	22.31	22.58	1.79	22.36	22.40	1.83	0.9408
WC [cm]	78.50	78.50	7.93	79.84	78.00	9.87	79.88	79.00	9.16	0.8275
NC [cm]	32.88	33.00	1.96	33.49	33.00	2.55	34.12	34.00	3.40	0.4150
Glucose [mg/dl]	97.43	94.00	22.73	89.13	86.90	16.95	90.06	89.00	15.24	0.6013
TG [mg/dl]	111.11	98.00	65.76	110.03	92.00	69.94	113.67	97.00	70.43	0.7111
HDL [mg/dl]	66.22	68.00	15.11	73.56	71.00	18.35	72.40	70.00	17.72	0.6950
AST [IU/l]	27.00	28.00	5.58	24.98	24.00	6.08	27.09	25.00	8.63	0.1661
ALT [IU/l]	26.50	23.50	6.21	22.05	21.00	8.01	25.90	22.00	13.71	0.0717
**Group with MS**										
Age [years]	61.50	66.00	13.25	59.77	63.00	12.16	60.22	63.00	11.87	0.6567
Body mass [kg]	77.23	79.00	9.85	83.05	82.00	14.60	81.25	80.00	15.04	0.3362
BMI [kg/m^2]	27.37	26.64	3.49	30.37	30.00	5.04	29.89	29.00	4.76	0.0660
WC [cm]	95.00	98.50	9.54	102.05	101.00	12.43	101.15	99.00	12.65	0.2544
NC [cm]	36.32	36.00	2.72	38.14	38.00	3.48	37.57	37.00	3.67	0.1755
Glucose [mg/dl]	106.10	102.00	20.32	107.36	99.00	36.05	107.25	100.00	31.98	0.9050
TG [mg/dl]	182.20	191.00	54.43	240.15	196.00	188.29	207.23	178.00	104.57	0.5130
HDL [mg/dl]	53.20	52.50	16.03	57.03	55.00	14.28	57.27	54.00	15.29	0.6321
AST [IU/l]	23.33	23.00	4.09	29.42	27.00	11.90	30.02	27.00	12.35	0.0913
ALT [IU/l]	27.89	24.00	13.92	35.22	29.50	19.13	35.65	30.00	20.68	0.2925
**Group without MS**										
Age [years]	57.56	58.00	12.15	50.36	52.00	13.92	50.79	52.00	14.57	0.0609
Body mass [kg]	74.83	75.00	12.33	68.99	66.50	13.41	72.31	69.50	14.83	0.0129[2]
BMI [kg/m^2]	26.60	26.70	4.22	25.21	24.20	4.29	25.83	25.30	4.40	0.0776
WC [cm]	91.50	94.00	12.30	86.20	85.00	13.38	88.77	88.00	13.94	0.1032
NC [cm]	36.32	36.50	3.55	34.89	35.00	3.47	35.86	35.00	3.87	0.0236[2]
Glucose [mg/dl]	90.83	91.50	10.30	87.58	87.00	13.23	88.11	87.00	14.20	0.2535
TG [mg/dl]	101.96	92.00	58.54	105.58	92.50	58.03	108.91	99.00	53.47	0.3128
HDL [mg/dl]	63.81	61.00	17.99	70.40	68.00	17.62	69.04	66.00	16.47	0.1009
AST [IU/l]	26.41	27.00	5.51	27.62	25.00	14.11	27.64	26.00	10.03	0.3588
ALT [IU/l]	26.91	23.00	9.99	29.03	23.00	23.18	29.18	26.00	20.98	0.1069

SD: standard deviation; BMI: Body Mass Index; WC: waist circumference; NC: neck circumference; TG: triglycerides; HDL: high density lipoprotein; AST: aspartate transaminase; ALT: alanine transaminase; MS: metabolic syndrome; Kruskal-Wallis test was performed and when $p<0.05$ post-hoc Dunn's test was applied. [1]The difference between TT and GT was shown for ALT (p=0.0419); [2]The difference between GT and GG was shown for body mass (p=0.0461) and NC (p=0.0398)

Table S7

Comparison of anthropometric measurements and metabolic parameters between particular genotypes of rs10423928 variant in the control and the study group, and the group with and without metabolic syndrome.

**rs10423928**	AA	AT	TT	p

	Mean	Median	SD	Mean	Median	SD	Mean	Median	SD	
**Study group**										
Age [years]	58.32	62.00	11.26	57.07	60.00	12.98	56.59	60.00	13.58	0.8188
Body mass [kg]	80.53	77.00	15.33	82.35	82.00	13.53	82.47	81.00	12.98	0.6333
BMI [kg/m^2]	29.25	27.70	4.20	29.46	28.40	4.05	29.94	29.00	4.06	0.1012
WC [cm]	97.93	96.00	12.27	99.18	98.00	12.04	99.68	99.00	11.88	0.4244
NC [cm]	37.74	37.00	3.65	37.68	38.00	3.64	37.57	37.00	3.47	0.9260
Glucose [mg/dl]	98.14	90.00	24.91	99.60	93.00	30.31	97.73	92.00	26.85	0.7656
TG [mg/dl]	205.94	156.00	135.74	172.24	148.00	111.81	165.60	136.00	125.23	0.1437
HDL [mg/dl]	60.69	59.50	13.37	59.83	60.00	14.74	60.90	59.00	15.94	0.9062
AST [IU/l]	29.72	27.50	9.25	29.29	26.00	14.98	29.65	27.00	12.16	0.2693
ALT [IU/l]	36.53	27.50	23.70	33.97	28.00	21.94	35.49	30.00	24.68	0.6000
**Control group**										
Age [years]	44.35	41.00	12.11	48.46	48.00	15.29	48.41	47.00	14.25	0.4279
Body mass [kg]	62.83	61.80	5.66	62.06	60.00	9.49	62.21	60.70	8.64	0.6269
BMI [kg/m^2]	21.82	21.80	1.73	22.37	22.31	1.82	22.37	22.60	1.80	0.2662
WC [cm]	76.84	77.75	6.27	80.40	79.00	10.36	79.73	79.00	8.96	0.3574
NC [cm]	34.44	34.00	2.69	33.67	33.00	3.18	33.81	33.00	3.01	0.4564
Glucose [mg/dl]	91.71	90.00	11.73	89.48	88.40	15.66	90.14	87.00	16.88	0.4054
TG [mg/dl]	111.61	81.50	67.27	119.57	97.00	80.17	106.94	92.00	61.92	0.5949
HDL [mg/dl]	77.11	79.50	21.41	72.42	70.00	18.08	72.46	69.50	17.00	0.7593
AST [IU/l]	27.00	23.00	9.97	26.97	25.00	9.11	25.85	25.00	6.50	0.9724
ALT [IU/l]	24.27	20.00	10.65	26.00	22.00	15.34	23.34	22.00	9.03	0.7016
**Group with MS**										
Age [years]	60.76	63.00	10.87	60.37	63.00	11.30	59.69	63.00	12.68	0.9667
Body mass [kg]	80.24	79.80	12.63	80.71	80.40	14.90	82.80	80.93	14.99	0.5016
BMI [kg/m^2]	28.89	27.50	3.94	29.55	29.20	4.72	30.42	29.80	4.97	0.0927
WC [cm]	99.50	97.50	10.83	99.90	100.00	12.61	102.47	100.50	12.49	0.1380
NC [cm]	37.70	37.00	3.50	37.55	37.00	3.79	37.90	38.00	3.46	0.6724
Glucose [mg/dl]	107.41	98.00	29.54	107.48	101.00	34.63	106.83	97.00	32.32	0.8814
TG [mg/dl]	256.29	178.00	149.54	215.25	186.00	122.36	217.10	192.00	150.23	0.7099
HDL [mg/dl]	58.67	54.00	15.68	57.96	54.50	15.43	56.01	54.00	14.36	0.5174
AST [IU/l]	30.95	26.50	10.95	27.10	25.00	8.64	31.39	28.00	13.93	0.0027[1]
ALT [IU/l]	42.40	28.50	28.99	31.77	27.00	16.59	37.17	31.50	20.55	0.0114[1]
**Group without MS**										
Age [years]	49.97	46.00	12.22	49.37	51.00	14.78	51.37	52.00	14.36	0.4057
Body mass [kg]	71.81	66.30	16.54	70.62	67.50	15.51	71.59	69.50	13.62	0.4601
BMI [kg/m^2]	25.75	24.23	5.30	25.14	24.75	4.29	25.90	25.30	4.26	0.1064
WC [cm]	86.38	82.50	14.54	87.18	88.00	14.36	88.55	88.00	13.27	0.2893
NC [cm]	36.10	35.00	3.85	35.43	35.00	3.94	35.52	35.00	3.65	0.7240
Glucose [mg/dl]	88.95	89.00	9.98	87.00	86.00	11.77	88.61	87.00	14.90	0.3903
TG [mg/dl]	122.42	108.00	68.75	105.65	97.00	52.58	106.36	97.00	54.12	0.5495
HDL [mg/dl]	70.21	62.00	18.37	69.29	66.00	16.65	69.14	67.00	16.79	0.9516
AST [IU/l]	27.17	25.50	8.02	29.73	26.00	16.49	26.64	25.00	7.98	0.5067

ALT [IU/l]	25.63	23.00	9.52	31.01	25.00	23.37	28.21	24.00	20.86	0.5923

SD: standard deviation; BMI: Body Mass Index; WC: waist circumference; NC: neck circumference; TG: triglycerides; HDL: high density lipoprotein; AST: aspartate transaminase; ALT: alanine transaminase; MS: metabolic syndrome; Kruskal-Wallis test was performed and when $p<0.05$ post-hoc Dunn's test was applied. [1]The difference between AT and TT was shown for AST ($p=0.0022$) and ALT ($p=0.0113$)
